# Performance Prediction Criteria Based on Yearling Training Cycle Data for World-Class Athletes’ Tiny 1000-Meter Kayak Teams: A Case Study

**DOI:** 10.3390/life15030476

**Published:** 2025-03-16

**Authors:** Stanislav Dadelo, Ričardas Nekriošius, Rūta Dadelienė

**Affiliations:** 1 Department of Entertainment Industry, Vilnius Gediminas Technical University, 10223 Vilnius, Lithuania; 2 Department of Coaching Science, Lithuanian Sports University, 44221 Kaunas, Lithuania; ricardas.nekriosius@lsu.lt; 3 Institute of Health Sciences, Vilnius University, 01513 Vilnius, Lithuania; ruta.dadeliene@mf.vu.lt

**Keywords:** double kayak team, pre-Olympic cycle, world-class athletes, physiological criteria, periodization

## Abstract

This research aimed to identify optimal performance needs based on physiological tests of kayakers, revealing body adaptations and critical training periods within a yearly training cycle. It sought to develop performance selection protocols for teams and provide evidence-based strategies for future training. Methods: The male athletes underwent routine physiological testing, considering medical limitations. A preparation year plan was established: six months for preparation, one month for the first competition, two months for further preparation, one month for the second competitor, and two months for transition. The athletes faced twelve tests at the start of each month. Results: A certain intensity and duration of training effort during preparatory cycles (approximately 22–42% in the third intensity zone, 24–28% in the fourth intensity zone, and 3–4% in the fifth intensity zone) were necessary for athletes to achieve a high level of aerobic metabolism (64.00 and 69.40 mL·min^−1^·kg^−1^). Heart rate, work capacity at the second ventilatory threshold, the critical intensity limit, and maximum oxygen consumption were all shown to be vital indicators for predicting competition results. Conclusions: The identified indicators of physical development and functional capacity could aid in forming a team capable of reaching an elite level in the future.

## 1. Introduction

A small sports team may consist of as few as 2 to 10 players [1]. Since each player’s performance greatly impacts a group’s success, small sports teams encourage candid communication, close teamwork, and a more cohesive dynamic [2]. Therefore, small team member selection and training planning should be conducted thoroughly. Small kayaking teams must be chosen and organized using a combined physiological, biomechanical, and psychological stress training approach [3] due to individual differences in power generation, physical characteristics, psychological dynamics, and paddling style.

It must be emphasized that a team training process’s administration must be solely dependent on the information given by its participants [4]. The primary studies on team training for sprint kayaking were conducted to maximize performance in physiology, biomechanics, nutrition, psychology, and training techniques [5]. Each of these domains holds significance, and issues are usually resolved independently. Comprehensive research in this field is lacking. In sprint kayaking, both aerobic power for powerful strokes and setting up continuous endeavors must be fused to the anaerobic aptitude of the racer [6]. This is done by enhancing maximum oxygen consumption (VO_2_max) [7]. The better balance position, the end of maintaining balance, and the forceful involvement of gross motor movement from the rowing position are significantly important during special training [8]. Combined with plyometrics, the key feature of training periodization refers to repetitions of high-load and low-resistance exercises [9]. Maintaining performance, particularly in multi-race events, depends partly on the ability and expertise to recover well [10].

Training sessions should be periodized to develop strength, endurance, and pace gradually. Imitations of competitions involve controlled HIT (high-intensity training), in which the exerciser performs short, intense bouts of exercise followed by recovery periods, which replicates race circumstances [11]. Cross-training prevents injuries that result from the repetitive use of certain muscle groups, not to mention that it helps keep the cardiovascular system strong [12]. Tools like heart rate monitors, GPS trackers, and power meters are part of an athlete’s training regimen and offer instant information about an athlete’s ability [13]. Team management includes focused instruction and strategies designed to build harmonious, balanced teams. The athlete training process algorithm explains the methodically planned procedure for improving performance using training formulation techniques while preventing overtraining or injuries [14]. The training progression and research findings are incorporated into a logical sequence of an athlete’s actions and understood as a training process algorithm. To achieve performance goals and minimize the risk of injury, the training algorithm seeks to ensure that an athlete is ideally prepared regarding training doses (volume, intensity, and recovery) [15]. The algorithm aids in optimizing the transition between demanding training sessions. Creating an algorithm aids in systematizing the training process and guarantees athletes’ recovery, ensuring that training is scientifically validated and adapted to changing circumstances.

Athletes must possess a range of physical abilities to satisfy these requirements and be successful in world-class double kayaking [16]. It is relevant to identify the markers of aerobic metabolism and regularly evaluate those [17]. Kayakers rely mostly on aerobic metabolism, which they maintain at an intensity of 85–90% of their aerobic capacity and almost the VO_2_max for most of a race [18]. The athlete preparation periodization technique involves allocating training durations and activity levels to produce the intended oxidation stresses [19]. It is relevant to creating protocols for high-intensity interval training (HIIT) [20]. HIIT exercises provide maximum aerobic and anaerobic advantages within short exercise sessions. The training includes repetitions of a maximal-effort exercise with low-intensity exercises; this is repeated a few times. HIIT’s aerobic and anaerobic energy expenditure provides superior workout benefits in less time than moderate-intensity continuous training (MICT), which also achieves these outcomes. Proper recovery strategies are needed to manage rapid exhaustion because of the high-intensity requirements of HIIT methods. HIIT training matches or exceeds traditional endurance training benefits while demanding less time from participants [21].

HIT describes a training system where exercise consists of brief, powerful efforts interspersed with recovery through rest or lower-intensity work. The sports science community has been more interested in defining training regimens that enable athletes to sustain prolonged durations beyond 90% of their VO_2_max, even if HIT is not the only strategy for enhancing physiological characteristics and performance [22]. The exercise method finds widespread use in general training practice because it effectively boosts cardiovascular endurance, strength, and metabolic capabilities. The time-efficient training technique HIT demonstrates maximum effectiveness as an exercise suitable for diverse athletes. To obtain the maximum possible benefits while reducing potential risks, proper recovery techniques and customized training protocols must be implemented. Many variations in HIT training present different intensity levels and specific duration and rest periods of work-to-rest ratios. Athletes who use HIT exercises perform at the highest intensity level for 20 s, followed by 10 s of rest over four minutes. Sprint Interval Training (SIT) includes 30 s of total effort sprints followed by 4–5 min of recovery time. Aerobic HIT involves longer intervals (e.g., 3–5 min) at submaximal intensity with shorter rest periods. The strength-based version of HIT combines intense resistance movements using minimal rest moments between sets.

Combined training is the most effective exercise intervention [23]. One study found that block periodization and traditional training did not significantly alter the endurance–training load [24]. During the preparatory phase, the small team’s one-year training cycle focused on balancing different training styles and the length and intensity of physical loads. Divergent views exist on the relative importance of certain aspects of the markers. Tønnessen et al. [25] focused on the functional metrics of athletes, while Rees et al. [26] examined anthropometric data. It is not apparent which basic guidelines should be followed when matching kayakers, which athletes’ traits should be as similar as possible, and which can not match to achieve certain goals and obtain more precise preparation forecasts.

Training a small kayak team and preparing for an annual cycle requires a methodical approach to creating a management algorithm that combines physical skill with race-specific training [27]. However, comprehensive research in this field is lacking. Still, world-class kayak athlete training programs derive methodology from targeted research studies investigating performance elements, including body morphology, strength development, and specialized training program structures [28,29]. Team preparation encompasses carefully measuring and adjusting preparation loads to achieve peak form during the competition season and maintaining physical and psychological preparedness for a world-class level of competition [30].

This research evaluated an entire year of 1000 m kayaking training by investigating the ideal combination of resistance training and kayaking performance exercises to optimize athletic results during elite competition. This research aimed to identify optimal performance needs based on physiological tests of kayakers to reveal body adaptations and critical training periods in a yearly training cycle, develop performance selection protocols for teams, and deliver evidence-based future training strategies.

## 2. Materials and Methods

### 2.1. Subject

A world-class team of two male flat-water kayak athletes (K2) (participant A was the front paddler and participant B was the back paddler) who were both Olympic 1000 m finalists and World Championship medalists agreed to participate in this investigation. They correspond to the world class athletes’ classification [31]. The individuals displayed the following traits: 184.5 and 186.0 cm tall, seven and eight years of experience training for international contests, and 29 and 30 years old. During 1000 m distance tests, the average time was 3 min and 20 s in the one-year training cycle. The athletes were informed about the experimental protocols and provided written agreement. This research was conducted according to the guidelines of the Declaration of Helsinki and approved by the Ethics Committee of Lithuanian Sports University (no. 158200-18/11-1040-573) on 6 November 2018.

### 2.2. Research Design

**Figure 1:**
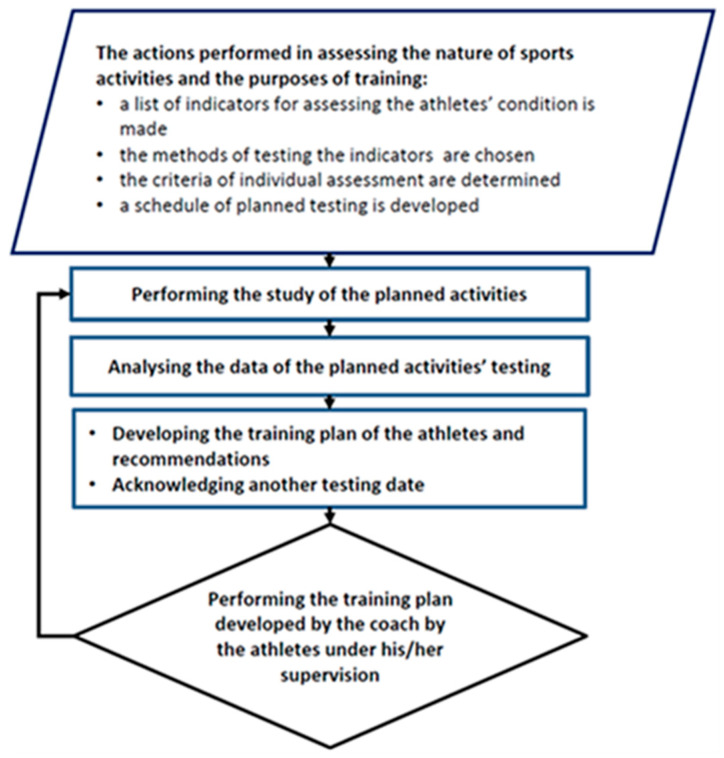
The management algorithm of the world-class athletes’ training process.

**Figure 2:**
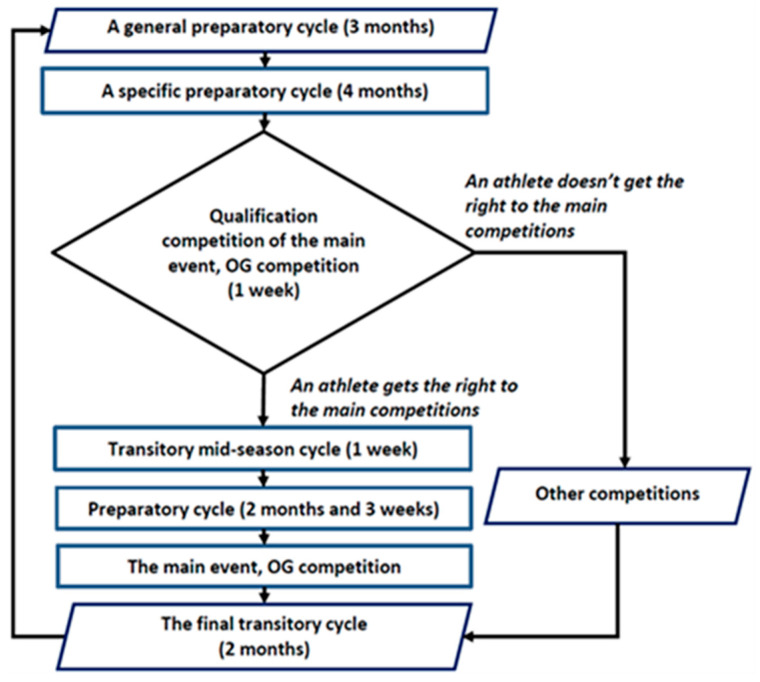
The algorithm of the one-year training cycle for the world-class athletes.

This study was carried out in the fourth year of the cycle when the athletes were preparing for the Olympics, and an approved algorithm (Figure 1) controlled their training regimen. Throughout the research, the male athletes received routine physiological testing with medical limitations. The athletes were to follow their training regimen precisely but not to exercise the day before tests. During the testing process, the athletes were not injured and did not feel pain. Every testing session began in the morning and was conducted for 9–11 h under consistent environmental conditions in the laboratory of the Sports Science Institute. A preparation year plan was set: six months for preparation, one month for the first competition, two months for the second preparation, one month for the second competition, and two months for the transfer (Figure 2). The athletes underwent twelve tests at the start of each month. November saw the first testing session and October of the following year saw the final one. The diet of the subjects was based on the recommendations of a dietitian. No dietary data were collected.

### 2.3. Training Monitoring

Throughout this research, a skilled coach controlled the process of training. The training activities were divided into groups: (1) specialized—performance on the water or using the Dansprint kayak ergometer; (2) nonspecific—gym resistance training. The applied training load was divided into five intensity zones: 1–2 (low intensity) and 3–5 (high intensity (HI)) [32] (Table 1). During the training session, team performance was tracked on the water and a Dansprint kayak ergometer (Denmark). The computer system “Garmin Connect Forerunner 910 XT” (Switzerland) was used to measure HR, boat speed (m/s), and training duration (h).

### 2.4. Variables and Testing Procedures

The athletes’ height (cm) was measured with a height meter SOEHNLE Professional. Body mass (kg), muscle mass (kg), and fat mass (kg) were evaluated using a body composition analyzer (Jawon Medical^®^ IOI-353-CE0197, Seoul, Republic of Korea). Both hands’ maximum hand grip strength (kg) was assessed by a hydraulic hand dynamometer, JAMAR, 5030J1, Warrenville, IL, USA [33]. Vital lung capacity (VLC) (diagnostic spirometer Micro I, Cardinal Health, Leeds, UK) was measured [34]. A personal computer, special computer program, and reactiometer RA-1 (JSC BALTEC CNC TECHNOLOGIES, Kaunas, Lithuania) were used to measure psychomotor reaction time (mls); participants pressed a button with their writing hand in response to a light signal [35]. Movement frequency was measured using a taping test per 10 s with the same equipment [36].

Heart rate (HR) (b/min) baseline at rest was measured after the subjects had been lying down and relaxing on a couch for 5 min in a quiet environment (+24 °C) with a telemetric HR monitor (Polar RS800 CX, Kempele, Finland). The concentration of Hemoglobin (g/L) and Hematocrit (%) from capillary blood was measured using a DiaSpect HB spectrophotometer (Sailauf, Germany). Mobile 781023-052, version 5.2 (Cardinal Health, Leeds, UK) was used to create submaximal ergometer tests. Before each test, the device was calibrated according to the manufacturer’s instructions. Using a kayak ergometer (Dansprint PRO, KE001 ergo, Hvidovre, Denmark), each participant completed the incremental submaximal ergometer test to ascertain VO_2_max [37]. Before the submaximal ergometer test, the participants warmed up for five to ten minutes and rested for five minutes. To get the athlete to the tolerance limit in 8–12 min, the incremental test started with a workload of 100 W and applied increments of 20 W at 30 s intervals. The results were averaged over 30 s intervals. Work capacity (W), oxygen consumption (VO_2_) (mL·min^−1^·kg^−1^), and HR (b/min) were measured at the critical intensity limit (CIL) at the second ventilatory threshold (VT2) methods [38].

### 2.5. Statistical Tools

The data collected throughout the study period were compared across the research subjects. They also evaluated how variable the indicators were during the research period. Indicators for data analysis included the standard deviation (SD), average (X), coefficient of variation (V), mathematical difference (d), test reliability (t), and Student *t*-test criterion values (*p* < 0.05, *p* < 0.01, *p* < 0.001).

## 3. Results

The qualifying Olympic Games events (held in May) comprised the first macrocycle, which included general and specific preparation; competitor cycles; and a one-week transient mid-season cycle. The preparation and competition cycles for the Olympic Games (in August) made up the second macrocycle (Figure 2). General training data for the full year of training features are displayed in Table 2.

The training load was computed to plan the athlete’s training volume and intensity. The techniques outlined by Foster et al. [39] were used to monitor the RPE (Rating of Perceived Exertion) training load to evaluate athlete performance and recovery. According to Barbero-Álvarez et al. [40], recommendations and training loads can be evaluated by combining RPE with GPS data. Training load was measured using Training Impulse (TRIMP) according to HR zones and session length [41]. This made it possible to control the athletes’ physiological reactions and intensity. Over the year, 239 sessions were conducted, totaling 691 h of training. The overall workload time climbed to 77 monthly hours throughout the preparation cycle. The Olympic Games were the most significant of the athletes’ 22 annual starts. Resistance training in a gym took 243 h, whereas kayak performance training took 448 h. During the first preparatory (general) cycle, the amount of work required for the kayak performance and the resistance training in the gym grew: 36.5% of the team performance training time was in low-intensity zones 1–2, while 57.5% was in HI zones 3–5, according to the distribution overview based on training time in various intensity zones (Table 3). 

The third intensity zone accounted for 41% of the overall training volume and had the highest work intensity (the primary area for the development of aerobic metabolism). Anaerobic metabolism increased in the fourth intensity zone (high physical intensity of 25%). During the general preparatory cycle, the athletes had higher glycolytic loads for 3% of the time. During specific preparation cycles, more time was spent generating glycolytic processes (4%) and activating maximal aerobic metabolism (26%). In April, the last month of the special preparatory cycle, the third intensity zone increased to 30%, the workload in the fourth intensity zone increased to 28%, and the burden in the fifth zone increased to 4%. The objective of raising the skill of intensity was connected to sustaining a high level of performance and fitness through the beginning of May. The competitor cycle, or Olympic qualifying event, had to happen between May 5 and May 8. After that, athletes were given seven days to rest. The second training cycle aimed to prepare competitors for the August 19–20 Olympic Games. The athletes’ workload was 27%, the fourth intensity zone’s workload was 25%, and the fifth zone’s workload was 4% of the total workout period, which increased the third intensity zone’s effort. The Olympic Games were successful (athletes participated in the final race).

**Figure 3:**
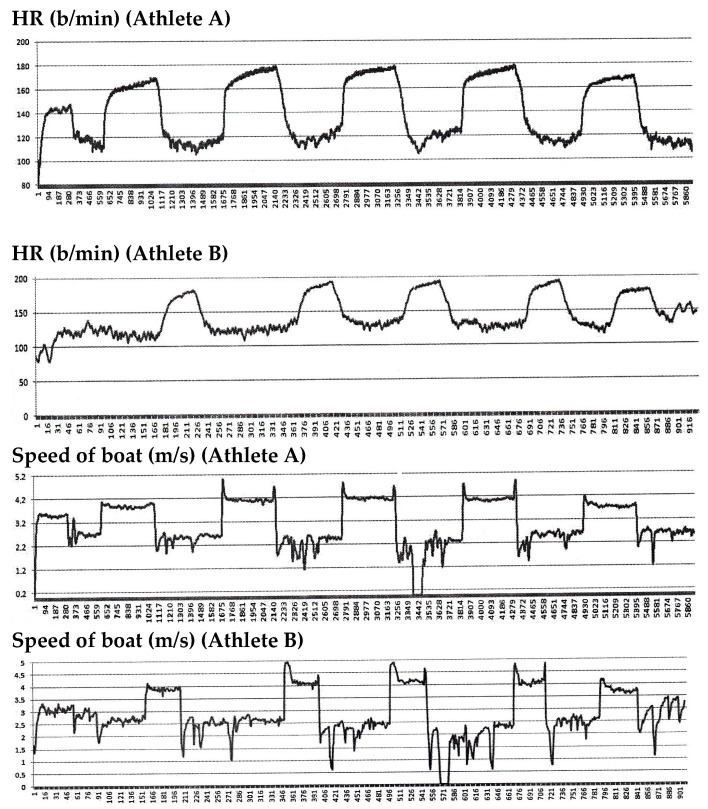
The data of athletes’ HR (b/min) and speed of the boat (m/s) during exercises (5 min × 6 times of maximal power).

The microcycle of the competition mesocycle with the highest training load was the microcycle of the training content, which was too high in physical effort (Table 4). Monday’s first interval training session aimed to strengthen the cardiovascular system. The goal of the second interval training exercise after the training session was to raise the creatine phosphate power. Monday’s second workout aimed to increase strength and capacity on the water. The goal of Tuesday’s workout was to increase glycolytic capability. Wednesday was a rest day. The goal of Thursday’s first session was to increase both anaerobic and aerobic endurance to sustain power. The end of the same workout was intended to increase power by promoting creatine phosphate reactions. Thursday’s second workout aimed to build more power on the water. Friday’s workout aimed to increase VO_2_max power and combined aerobic and anaerobic capability. Saturday’s training aimed to build power, endurance, heart performance, and circulatory system capability. Sunday was for the recuperation and super compensation of athletes. Variations in the boat’s rate and speed throughout one of the rowing exercises (maximum power for 5 min on the water; 6 min rest periods) (Figure 3).

During this exercise, there were comparable patterns in the athletes’ heart rates and the boat’s speed. Table 5 shows a minor change in the athletes’ body indicators during the Olympic year training program. Athletes’ body masses ranged from 87.5 to 89.6 kg and 83.0 to 85.5 kg. Before the Olympic Games, their muscle mass was between 49.1 and 46.9 kg. The ranges for fat mass were 7.1–7.6 kg and 6.4–7.8 kg. Psychomotor reaction times over one-year training cycles were 156–167 and 161–168 mls, whereas their movement frequencies were 77–86 and 78–105 times per 10 s, respectively. The resting heart rates of athletes A and B were 48.17 ± 5.01 b/min and 53.00 ± 3.86 b/min, respectively, over the annual training cycle and 12 testing sessions. However, the CIL HR data showed that athlete A’s was 183.92 ± 3.80 b/min and athlete B’s was 193.33 ± 7.62 b/min. There was a trend to raise the VO_2_max results, according to the athletes’ aerobic metabolism analysis during the one-year training cycle. The differences were 53.4–69.4 mL·min^−1^·kg^−1^ for athlete B and 57.7 to 68.6 mL·min^−1^·kg^−1^ for athlete A. At the time of the most significant starts, working capacity and VO_2_ indicators at VT2 and CIL peaked for the training year. Both athletes’ labor capacities were 260 W at VT2 and 340 W at CIL. These adjustments were sufficient to advance to the Olympic final race. For athletes to recuperate more thoroughly, the transitory cycle was necessary. It was not practical to abandon control of the functional and physical conditions. This study found that the athletes maintained their muscle mass during the brief cycle. VT2 and CIL’s work capacity, however, declined. Indicators, such as heart rate, hemoglobin concentration, etc., were found to differ across athletes on the same team. Individual differences allowed for a better adjustment of the physical burden of this squad. Additionally, it was demonstrated that VO_2_max, work capacity at CIL, and VT2 were all extremely significant indications that coincided.

## 4. Discussion

The researched athletes put in 691 h of training annually, including 448 h of kayak performance training. According to recommendations [25], athletes train for about 800 h a year, with about 500 h dedicated to training for a special activity. The kinematic markers and the loading of their cardiovascular and muscular systems in this study varied between training sessions (using a Dansprint kayak ergometer) and kayaking on the water [39]. Supercompensation in sports training ability development cycle processes is crucial, especially in high-performance disciplines [40].

A small kayak team may perform high-speed intervals (e.g., 500 m intervals) as part of their training regimen for a 1000 m race. To recover, a team needs a certain amount of time to rest; for example, the studied athletes had 9 days monthly during preparatory and competition periods and 14–15 days of rest in the transitory period. If the rest time is optimal for athletes, it is possible to increase the intensity or distance in each phase of the training process, helping athletes achieve their limits and causing adaptive responses. It is important to observe the cycle of stress and recovery in training as it can have different effects under management. Implementing physical training sessions is necessary for strength and endurance, necessitating a balance between stimulation and rest. This is crucial because supercompensation happens during recovery. Thus, training must be difficult enough to encourage adaptation [20].

It is important that training loads be planned taking into account the unique demands of the race course as well as the traits of each athlete. Kayakers must paddle for most of a race at or near-peak VO_2_max due to the sport’s characteristics [18]. This indicator is highly informative regarding kayakers’ preparation for a 1000 m race. To improve VO_2_max, the training effort’s volume and intensity are crucial; these two factors must be carefully correlated during the planning phase, which is based on determining the speed at VO_2_max. However, other factors could influence paddlers’ performance besides measuring their maximum oxygen uptake. Depending on the volume, intensity, and complexity of the effort, as well as the athlete’s training level, aerobic training raises VO_2_max. Our study shows that during the yearly Olympic cycle, VO_2_max gradually increased and reached the maximum level during (athlete B) and after (athlete A) competition. A good program design helps enhance the VO_2_max and should involve moderate to high volume and high-intensity intervals [41].

The athletes worked out with a kayak ergometer for 64 h during the general preliminary cycle. Then, they worked with a kayak on the water for 125 h during the special preparatory cycle, spending 28% of their time in the VT2 zone. The level of performance and fitness peaked after the preparation macrocycle. At VT2 and CIL, the aerobic (work) capacity significantly increased. VO_2_max attained the levels that previous authors have reported [42]. Similar to the data, the subjects’ mean VO_2_max value after the training cycle was between 54 and 60 mL·min^−1^·kg^−1^ [18]. Nonetheless, the athletes under consideration achieved mean VO_2_max values of 67.8 and 68.1 mL·min^−1^·kg^−1^ [43] but fell short of that reported by Lundgren et al. [44], which came in at 73.7 ± 6.3 mL·min^−1^·kg^−1^. Additionally, the VO_2_max for the world-class kayakers was approximately 58 mL·min^−1^·kg^−1^ [45] and 53.8 mL·min^−1^·kg^−1^ [42]. Much focus was dedicated to increasing the VO_2_max during training, although it cannot surpass a specific biologic–hereditary limit, regardless of training duration and intensity.

Monitoring key endurance and HIT aerobic and anaerobic efficiency metrics is crucial for managing sprinter canoeists’ training development [46]. The highest effort put out during physical activity is reflected in the CIL [47]. It serves as a standard for evaluating anaerobic thresholds and training loads. The ideal measure of respiratory and cardiovascular fitness is VO_2_max [48]. It is strongly linked to the endurance feature and enables the measurement of the maximum oxygen consumption rate during vigorous physical activity. However, several coaches and scholars [49,50] have questioned its importance. Nonetheless, most top endurance athletes report VO_2_max levels that are exceptionally high. However, top athletes rarely succeed in endurance sports without strong aerobic capacity. VO_2_max is roughly equal to 69 mL·min^−1^·kg^−1^ for top athletes, indicating excellent aerobic efficiency. The VT2 work capacity indicator indicates how effectively an athlete’s body can withstand lactate generation after prolonged exertion and provides information about the threshold limitations while transferring from aerobic to anaerobic metabolism [51]. Research shows that these factors are crucial for improving power and endurance during rowing sprint training. By monitoring these physiological indicators during training cycles and adhering to basic preparation principles, athletes can predict how well they will compete.

Frequently performed aerobic exercises due to alterations of such factors as stroke volume, capillary density, mitochondrial one, and the efficiency of neuromuscular junction effect VO_2_max at low intensity and shorter training sessions. Repetition is the first method and gradually increasing the load’s volume is the second to keep the cardiovascular and muscular systems under constant pressure [52]. After nine to ten weeks of training, there is a noticeable increase in VO_2_max, which varies from 15 to 25%. After that, it continues to rise, albeit at a slower rate. One important indicator of aerobic capacity is the lactate threshold. World-class kayakers may experience a lactate threshold between 81% and 91% of their VO_2_max, translating to power generation between 132 and 226 W [53]. Our study shows that athletes demonstrated 200–260 W of work capacity in VT2 and 320–340 W of work capacity in CIL. An athlete’s aerobic metabolism is determined by their greater artery diameters, their muscles’ capacity to consume oxygen, and how quickly their aerobic metabolism develops from the beginning of work. The cardiovascular system’s functional capacity affects oxygen intake, muscle oxygenation kinetics, and competition distance time. A high VO_2_max indicator influences an improved rate of tempo drop delay and speed persistence by competition distance time [54]. It also improves oxygen intake, which speeds up oxygen exchange between muscles. Therefore, in long endurance races, the cardiovascular system’s performance is crucial for sports success. Given the power required for speed, the anaerobic model component is crucial for kayaking, particularly in the sprinting category [55].

The anaerobic system is also the most suitable energy supply model for kayak sprinting due to its brief duration and high power generation under stress and competition conditions. However, aerobic endurance is also essential, especially in distance races. Therefore, anaerobic training must prepare athletes to sustain the necessary speed and regulate lactate buildup throughout HIT [18]. Since kayakers’ performance in 1000 m races depends on aerobic and anaerobic (phosphocreatine and glycolytic) metabolism, great training efforts were made to stimulate glycolytic processes. Of the entire training time during the special preparatory cycle, 26% was spent in a mixed aerobic–anaerobic zone and 4% was spent in an environment that maximized the support of aerobic metabolism and activated glycolytic processes. This ratio reflects the rationale for skill and performance development in the training of top kayakers. Thus, water sessions may include interval training or long-distance paddling to simulate race conditions. Non-specific training, such as resistance exercises in the gym (e.g., pull-ups, squats, or ropes), builds muscle and core stability. Such guidelines reflect the rules and regulations of training periodization [56]. Different training periodization models give different results. Nonlinear and block periodization is better than traditional linear methods, especially for top athletes. This is due to training’s greater variability, which keeps performance from stabilizing and maintains advancement [57]. Periodization should be customized to the athlete’s goals, training level, and competition schedule for optimal results [58].

The training plan under consideration aimed to keep the athletes’ maximal aerobic metabolism and work capacity at CIL throughout competition cycles. Consequently, the training load and intensity zone distribution throughout the competition cycle were the same as during the specific macrocycle and preparatory cycle. Athletes cannot maintain high capacity for prolonged terms due to the physiological limits of energy production and the buildup of fatigue byproducts like lactate. However, proper training and pacing help optimize performance by maximizing high and sustainable intensities [59]. Two mesocycles made up the transitory cycle of the training regimen in question. This reduced both the workload and the overall length of training time. The aerobic metabolism markers were much lower at CIL during this cycle. They considered objective and subjective elements regarding process management and athlete performance expectations. Likewise, individual parameter variabilities like muscle mass, grip strength, heart rate, Hemoglobin concentration, and Hematocrit are common among world-class kayakers [60]. These differences reflect each athlete’s unique physiological and genetic makeup and body adaptation to training. In kayaking, this variability influences selection, team dynamics, and performance outcomes, as it is often impractical to find athletes with identical profiles to form a uniform team. Having a variety of physical and physiological traits among team members can benefit a sprint kayak team. The diverse capabilities of participants enable the team to handle different race demands and adapt to varying strategies. Therefore, it is important to harmonize these strengths through the training process and synchronized techniques to maximize a team’s performance [61]. However, in this study, psychomotor reaction time, VO_2_max, VO_2_, and work capacity at VT2 were the critical indicators for predictors. This provides a comprehensive picture of a kayak athlete’s physical endurance, psychomotor sharpness, and adaptability to intense competitive conditions. All these parameters act as fitness indices for optimal performance and victory because they assess the ability of an athlete to sustain high levels of strain for a long time, make timely adjustments, and recover during a race.

Limitations: Low statistical power occurs when a study selects a small sample size. The concept presented here may not support managing broader or more complex team sizes. The conclusions and suggestions for improvement presented here may not apply to various teams, performances, or genders. This research concerns only world-class athletes in this sport. Small team characteristics, for example, the relationships between team members and/or individual tendencies, may influence learning and performance.

## 5. Conclusions

Organizing an entire yearly training process in this study was a success for elite competitors with unique physical abilities. This study described how training should be spread between kayak-specific performance activities and gym-based resistance activities, with ratios ranging from 62 to 69% and 31 to 38% during pre-competition stages and competition periods. This study defined precise HIT zone allocations for peak performance preparation in 1000 m kayakers: 28% in zone 3, 25% in zone 4, and 4% in zone 5. Structured intensity distribution was verified by scientific research and confirmed to enhance aerobic capacity and readiness for important competitions. This research identified four key physiological parameters as crucial for monitoring world-class kayakers’ progress and competition outcome predictions: HR and work capacity at VT2 and CIL and VO_2_max. The findings indicate that these aerobic indicators were close to each other among the elite athletes, reinforcing their importance in training assessment and selecting young athletes. This study demonstrated the effectiveness of the annual cycle training program, as athletes started the competition successfully and took prize places in the Olympic Games. This study demonstrates that world-class kayaking requires psychomotor reaction time for predicting team compatibility and competition preparedness. The research findings about psychomotor reaction time could be a tool for athlete selection in small teams across multiple sports. Future research directions are essential to investigate how kayakers with diverse physiological profiles can achieve optimal performance. Researchers must find approaches that control micro and macrocycles to preserve top aerobic capacity. The selection of athletes should be based on physiological tests and the needs of the current sport. Data from physiological models of elite athletes, statistical evaluation methods, and multidimensional decision systems could help with the selection and management of training programs. This study brings forth enhanced knowledge regarding three key aspects of 1000 m kayaking training, including the distribution of training intensities and athlete monitoring systems. The research results provide implementation strategies for elite kayak physical and functional development ideas for training, planning, and preparation aspects for competitions.

## Figures and Tables

**Table 1:** Intensity zones for anaerobic and aerobic development by Seiler [32].

HIT Zones	HRmax
1	<60%
2	60–69%
3	70–79%
4	80–90%
5	>90%

**Table 2:** The workload of world-class kayakers (1000 m) in the final year of the Olympic cycle.

Macrocycles		I							II			Transitory		Workload (total)
Cycles		Preparatory General		Preparatory Specific				Competitor	Preparatory		Competitor			
Mesocycles		November	December	January	February	March	April	May	June	July	August	September	October	
Number of training days (n)		20	22	22	20	22	21	22	22	22	22	16	16	247
Number of training sessions (n)		22	26	28	32	34	36	34	34	34	28	16	15	239
Training time (h)	In the hall	24	26	30	25	25	21	20	20	16	20	10	6	243
	Rowing	28	36	40	40	45	56	45	40	40	40	20	18	448
Total training time (h)		52	62	70	65	70	77	65	60	56	60	30	24	691
Rowing (km)		252	-	-	121	383	536	515	560	463	415	180	270	3695
Number of competitions (n)		0	0	0	0	0	1	2	2	1	2	1		9
Starts (n)		0	0	0	0	0	1	6	3	4	6	2		22
Tests (n)		1	1	1	1	1	1	1	1	1	1	1	1	12

**Table 3:** Workload intensity of the world-class kayak athletes (1000 m) in the last year of the Olympic four-year cycle.

Macrocycles		I							II			Transitory		Workload (total)
Cycles		Preparatory General		Preparatory Specific				Competitory	Preparatory		Competitory			
Mezocycles		November	December	January	February	March	April	May	June	July	August	September	October	
**HIT Zones** % of kayaking performance time	**1**	10	16	16	22	23	20	27	29	21	20	36	21	17.0
	**2**	24	13	20	27	23	18	21	18	20	28	22	21	19.5
	**3**	40	42	36	22	25	30	25	27	27	28	29	32	30.25
	**4**	24	26	25	25	25	28	23	22	28	28	12	23	24.00
	**5**	2	3	3	4	4	4	4	4	4	4	1	2	3.25

**Table 4:** Example of content of microcycle training (in competition mesocycle) intended for developing high special physical load.

Week Days	Training Content
I	**1. Workout** 1. Warm-up: 20 min 2. Interval training: (a) 5 × (20 min, speed 60 s, La 4–6 mmol/L), rest breaks 5 min (b) Exercises: 10 × 15 s, rest break 2–3 min 4. Recovery exercises: 10–15 min
	**2. Workout** 1. Warm-up: 10 min 2. Exercises: (5 × 15–20 s) × 5, rest breaks 1–2 min 3. Rowing for 30 min (make a maximal powerful rower) 4. Recovery exercises: 10 min
II	1. Warm-up: 20 min 2. Repeated training (200 m with start, competition pace × 4, rest breaks 1 min, La 11–13 mmol/L) × 4, rest breaks 15–20 min 3. Steady rowing for recovery: 20–30 min 4. Recovery exercises: 10–15 min
III	Recovery–supercompensation.
IV	**1. Workout** 1. Warm-up: 20 min 2. Repeated training (2 km, La 5–6 mmol/L) × 4, rest breaks 5–8 min 3. Exercises: 10 × 15 s, rest breaks 2 min 4. Recovery exercises: 10–15 min
	**2. Workout** 1. Warm-up: 10 min 2. Exercises: (5 × 20–30 s) × 5, rest breaks 1–2 min 3. Rowing for 5 min × 6 (make maximal powerful rower), rest breaks 6 min 4. Recovery exercises: 10–15 min
V	1. Warm-up: 20–30 min (perform accelerations) 2. Repeated-control training (500 m × 2, rest breaks 3 min, La 8–10 mmol/L, rest breaks 15 min + 1 km, La 10–12 mmol/L) rest 20 min and 500 m × 2, rest breaks 3 min, La 10–12 mmol/L 3. Rowing for recovery: 20 min 4. Recovery exercises: 15 min
VI	1. Warm-up: 20 min 2. Interval training (20 min max fast 60 s, La 4–5 mmol/L) × 3, rest breaks 5 min 3. Exercises: (5 × 30–40 s) × 5, rest breaks 1 and 3 min 4. Recovery exercises: 10 min
VII	Recovery–supercompensation

**Table 5:** Physical development and aerobic metabolism data of the world-class kayak athletes in the last year of the Olympic four-year cycle.

Data		Month	BM, kg	Hand Grip, (R, L), kg		VLC	MM, kg	FM, kg	PRT, mls	FOM t/10 s	HR Rest b/min	Hb g/L	Ht %	HR, t/min CIL	VO_2_max mL·min^−1^·kg^−1^	WC, W, CIL	HR t/min VT2	VO_2_ mL·min^−1^·kg^−1^ VT2	WC, W, VT2
**Athlete A**																			
First macrocycle	General prep.	11	89.3	73	71	7.5	48.3	7.7	155	86	48	134	39	182	57.2	320	165	41.7	220
		12	87.5	70	68	7.7	47.5	7.1	165	80	48	158	46	181	60.9	320	164	43.5	200
	Specific prep.	1	88.0	68	66	7.5	48.0	7.3	151	81	46	163	48	183	62.2	340	170	51.9	240
		2	88.0	68	70	7.7	47.1	7.4	150	80	48	157	48	180	60.1	300	168	42.8	200
		3	87.5	70	71	7.6	47.1	7.2	157	77	48	164	48	183	60.7	320	165	42.0	220
		4	88.0	69	71	7.5	47.4	7.8	149	82	44	156	45	187	57.7	320	164	41.1	200
	Competition	5	88.5	69	65	7.6	46.7	6.8	155	82	44	167	49	184	61.8	320	166	49.5	200
Second macrocycle	Preparation	6	86.5	70	70	7.6	47.3	7.1	167	79	40	145	42	185	57.8	320	171	50.7	260
		7	89.0	68	68	7.5	49.1	7.6	162	79	52	145	42	184	64.0	320	169	50.8	240
	Competition	8	88.5	71	66	7.6	48.1	7.7	154	80	48	165	48	181	62.0	320	162	44.2	220
	Transitory	9	89.6	71	68	7.5	49.1	7.6	155	83	60	154	48	192	68.6	340	183	54.7	240
		10	89.0	72	72	7.6	48.3	7.6	155	81	52	161	53	185	58.8	320	165	43.6	220
** $\bar{X}$ **			**88.28**	**69.92**	**68.83**	**7.58**	**47.83**	**7.41**	**156.25**	**80.83**	**48.17**	**155.75**	**46.33**	**183.92**	**60.98**	**321.67**	**167.67**	**46.38**	**221.67**
**SD**			**0.88**	**1.62**	**2.33**	**0.08**	**0.78**	**0.31**	**5.69**	**2.29**	**5.01**	**9.88**	**3.80**	**3.23**	**3.18**	**10.30**	**5.53**	**4.77**	**19.92**
**V**			**1.00**	**2.32**	**3.38**	**1.00**	**1.63**	**4.17**	**3.64**	**2.83**	**10.39**	**6.34**	**8.20**	**1.76**	**5.21**	**3.20**	**3.30**	**10.28**	**8.99**
**Athlete B**																			
First macrocycle	General prep.	11	84.5	72	76	6.2	47.0	6.8	162	104	48	162	47	198	62.7	320	184	51.2	220
		12	84.0	89	81	6.3	46.2	6.6	161	90	56	180	50	202	55.7	320	192	45.8	200
	Specific prep.	1	84.0	90	82	6.2	46.3	6.7	156	95	60	171	47	199	53.4	320	184	44.4	200
		2	85.5	83	80	6.3	47.7	7.7	155	93	60	170	50	196	64.8	320	183	50.3	220
		3	83.0	84	74	6.3	45.7	6.4	151	90	48	179	52	196	64.3	300	180	47.8	220
		4	84.5	82	79	6.2	46.7	7.2	151	85	52	175	51	204	63.4	320	180	43.2	180
	Competition	5	83.0	90	81	6.3	47.4	7.8	154	*90*	52	163	48	206	62.2	320	190	48.2	200
Second macrocycle	Preparation	6	84.5	76	76	6.3	47.1	7.2	158	92	52	167	49	202	59.1	320	196	53.2	260
		7	83.5	76	74	6.3	46.1	6.8	155	85	52	163	48	193	63.9	320	184	55.6	240
	Competition	8	83.2	70	70	6.4	46.9	7.0	161	105	52	173	50	204	69.4	320	197	60.9	240
	Transitory	9	84.0	72	62	6.5	47.0	6.5	159	95	52	180	53	179	58.5	340	159	41.1	220
		10	84.5	71	74	6.4	47.2	6.7	168	78	52	177	54	189	65.1	320	179	47.1	200
** $\bar{X}$ **			**84.02**	**79.58**	**75.75**	**6.31**	**46.78**	**6.95**	**157.58**	**91.83**	**53.00**	**171.67**	**49.92**	**197.33**	**61.88**	**320.00**	**184.00**	**49.07**	**216.67**
**SD**			**0.75**	**7.68**	**5.67**	**0.09**	**0.59**	**0.45**	**4.94**	**7.61**	**3.86**	**6.76**	**2.27**	**7.62**	**4.45**	**8.53**	**9.98**	**5.58**	**22.29**
**V**			**0.89**	**9.65**	**7.49**	**1.43**	**1.26**	**6.45**	**3.14**	**8.29**	**7.29**	**3.94**	**4.56**	**3.86**	**7.19**	**2.67**	**5.42**	**11.37**	**10.29**
d			4.27	9.67	6.92	1.27	1.06	0.46	1.33	11.00	4.83	15.92	3.58	13.42	0.89	1.67	16.33	2.69	5.00
t			12.80	4.27	3.91	37.37	3.75	2.92	0.61	4.79	2.65	4.61	2.80	5.62	0.56	0.43	4.96	1.27	0.58
*p*			<0.001	<0.01	<0.01	<0.001	<0.01	<0.05	-	<0.001	<0.05	<0.001	<0.05	<0.001	-	-	<0.001	-	-

Note: body mass (BM), hand grip left (L), right (R), vital lung capacity (VLC), muscle mass (MM), fat mass (FM), psychomotor reaction time (PRT), frequency of movement (FOM), heart rate in rest position (HR rest), hemoglobin (Hb), hematocrit (Ht), heart rate at critical intensity limit (HR at CIL), maximal oxygen consumption (VO_2_max), work capacity at critical intensity limit(WC CIL),hear rate at econd ventilatory threshold (HR VT2)), oxygen consumption at second ventilatory threshold (VO_2_ at VT2), work capacity at second ventilatory thesthold (WC VT).

## Data Availability

The data used in this study are available upon request by the corresponding author.
